# Evaluation and simulation of nitrogen mineralization of paddy soils in Mollisols area of Northeast China under waterlogged incubation

**DOI:** 10.1371/journal.pone.0171022

**Published:** 2017-02-07

**Authors:** Yuling Zhang, Wenjing Xu, Pengpeng Duan, Yaohui Cong, Tingting An, Na Yu, Hongtao Zou, Xiuli Dang, Jing An, Qingfeng Fan, Yulong Zhang

**Affiliations:** 1 College of Land and Environmental Science, Shenyang Agricultural University, Shenyang, China; 2 Key Laboratory of Northeast Arable Land Conservation (Northeast China), Shenyang, China; Pacific Northwest National Laboratory, UNITED STATES

## Abstract

**Background:**

Understanding the nitrogen (N) mineralization process and applying appropriate model simulation are key factors in evaluating N mineralization. However, there are few studies of the N mineralization characteristics of paddy soils in Mollisols area of Northeast China.

**Materials and methods:**

The soils were sampled from the counties of Qingan and Huachuan, which were located in Mollisols area of Northeast China. The sample soil was incubated under waterlogged at 30°C in a controlled temperature cabinet for 161 days (a 2: 1 water: soil ratio was maintained during incubation). Three models, i.e. the single first-order kinetics model, the double first-order kinetics model and the mixed first-order and zero-order kinetics model were used to simulate the cumulative mineralised N (NH_4_^+^-N and TSN) in the laboratory and waterlogged incubation.

**Principal results:**

During 161 days of waterlogged incubation, the average cumulative total soluble N (TSN), ammonium N (NH_4_^+^-N), and soluble organic N (SON) was 122.2 mg kg^-1^, 85.9 mg kg^-1^, and 36.3 mg kg^-1^, respectively. Cumulative NH_4_^+^-N was significantly (*P* < 0.05) positively correlated with organic carbon (OC), total N (TN), pH, and exchangeable calcium (Ca), and cumulative TSN was significantly (*P* < 0.05) positively correlated with OC, TN, and exchangeable Ca, but was not significantly (*P* > 0.05) correlated with C/N ratio, cation exchange capacity (CEC), extractable iron (Fe), clay, and sand. When the cumulative NH_4_^+^-N and TSN were simulated, the single first-order kinetics model provided the least accurate simulation. The parameter of the double first-order kinetics model also did not represent the actual data well, but the mixed first-order and zero-order kinetics model provided the most accurate simulation, as demonstrated by the estimated standard error, F statistic values, parameter accuracy, and fitting effect.

**Conclusions:**

Overall, the results showed that SON was involved with N mineralization process, and the mixed first-order and zero-order kinetics model accurately simulates the N mineralization process of paddy soil in Mollisols area of Northeast China under waterlogged incubation.

## Introduction

Nitrogen (N) mineralization is not only a foundational step in soil organic N transformation, but it is also one of the most important processes in soil N cycling. More than 50% of N that is absorbed during rice growth is from the soil, regardless of whether or not N fertilizer has been applied [1, 2]. In fact, approximately 50%–80% of N is absorbed from the soil by high-yield rice [1]. During the N mineralization process, soil organic N is mineralised into inorganic N primarily in the forms of ammonium N (NH_4_^+^-N) and nitrate N (NO_3_^−^-N), or is transformed into an intermediate transitional fraction as soluble organic N (SON), which is then mineralised into inorganic N. Therefore, SON could be an available N source for plants. Historically, most studies on paddy soil N mineralization have focussed on soluble inorganic N and little attention has been paid to SON in leachates [3–6]. Therefore, a more accurate simulation of the N mineralization process in paddy soil is important to guide strategies for the rational application of fertilizer in rice production.

There is an extensive number of studies about the relation between N mineralization and soil properties. Soil organic carbon, TN, pH and texture could affect N mineralization [7–10]. During the process of organic nitrogen converting into inorganic nitrogen through microbial mineralization, the C/N value affects the ability of microbial decomposition [11], while pH and texture mainly affect the community and activity of microorganism [12–15]. Soil organic N (SON) and sand were relatively effective predictors of N_0_, which explained the variability of 40 and 34%, respectively [16]. Particulate organic matter N (POM-N) and pH explained 18% and 25%, respectively, of the variability in the first 2 weeks of aerobic incubation. Moreover, Abadín, González-Prieto [17] studied 21 soil samples and pointed out that H^+^, Ca^2+^, K^+^ of cation exchange capacity (CEC), the sum of CEC-bases (S_CEC_), total CEC (T_CEC_) and soil δ^15^N value are closely correlated with nitrogen mineralization, respectively, after aerobic incubation 3 weeks and 6 weeks.

Soluble organic N is a labile source of N for microorganisms, and some plant species (with or without associated mycorrhiza) can directly absorb simple organic N (e.g., amino acids) present in the SON pool [18–22]. The soil extractable organic N in water or salt solution is typically defined as soil SON, and as the SON pool in soils cannot be measured directly by extraction, it is instead determined by subtracting the mineral N concentration from the total soluble N (TSN) concentration [23, 24]. In a study of 12 agricultural soils of England, Murphy, Macdonald [23] found that the SON extracted with KCl solution accounted for 40%–50% of TSN. Soluble organic N is one of the active ingredients of soil organic N in incubation leachates [25, 26]. However, potential errors exist in evaluating soil N mineralization under aerobic incubation if the SON in leachates is not taken into account [25]. The conversion of insoluble organic N to low-molecular weight SON mostly limits the supply of soil N [27], and therefore the contribution of SON to soil N mineralization must be considered in the N mineralization process [28]. Lu, Li [29] found that the proportion of SON to TN was low under aerobic incubation conditions of ten major types of upland agriculture soil on the Loess Plateau, China. However, the SON pool was equivalent to the inorganic N pool in leachates under anaerobic incubation [30], indicating that TSN could partially reflect soil mineralisable N when SON is included under waterlogged incubation. Zhao, Cai [31] evaluated 12 types of agricultural and forestland soil on the Loess Plateau in China that were incubated under aerobic conditions, and found that measuring only the mineral inorganic N content could lead to an underestimate of the soil N mineralization potential and N loss. Therefore, the N potential mineralization capacity under waterlogged incubation includes inorganic N, as well as SON.

In order to describe the process of N mineralization well, several models have been proposed. Stanford and Smith [32] used a one-component, first-order kinetics model to simulate the mineralization dynamics during long-term aerobic incubation. Thereafter, other kinetics models were used, including the double first-order kinetics model [33–35], and the mixed first-order and zero-order kinetics model [3, 34, 36]. These models are widely used because they are simplistic, yet maintain sufficient theoretical foundation; additionally, they have been specifically applied to N mineralization studies of paddy soil [3, 6, 30].

The study of N mineralization of paddy soils in China is important, but there is little research on SON in N mineralization of these soils. When modelling the N mineralization process in paddy soil, most researchers limit their focus to inorganic N formed during mineralization, but do not fully consider the intermediate transitional SON that formed during mineralization. How would the N mineralization process model, N potential mineralization capacity, and N mineralization rate change if SON was included during the N mineralisation process? This question deserved further research and discussion.

In this study, N mineralization and simulation of paddy soils in Mollisols area of Northeast China were studied during 161 days of waterlogged incubation. The objectives of this study were (1) to evaluate the process and characteristics of organic N mineralization of paddy soils in Mollisols area under waterlogged incubation with or without SON in leachates, and (2) to compare the single first-order kinetics, double first-order kinetics, and mixed first-order and zero-order kinetics models, and to determine which model was the most suitable for describing the N mineralization process with or without SON.

## Materials and methods

The study was approved by the government of Qingan and Huachuan counties, which are located in Mollisols area of Northeast China. All experimental procedures were conducted in Shenyang Agricultural Univeristy.

### The soils

Soil samples were collected from the counties of Qingan and Huachuan, which are located in Mollisols area of Northeast China. The government of Qingan and Huachuan counties gave us permission to conduct this study on the field. These regions have long rice cultivation histories and large paddy fields that produce rice of high quality. Qingan County (127°30′–128°35′ E, 46°30′–47°35′ N) is located in the middle of Heilongjiang Province, and belongs to the upper reaches of the Hulan Basin on the Songnen Plains. This area is characterised by a temperate, semi-humid continental monsoon climate, a cold, long winter, mean annual temperature of 1.69°C, mean annual precipitation of 577 mm, and a 128-day frost-free period. Huachuan County (130°15′–131°34′ E, 44°34′–47°14′ N) is located in the east Heilongjiang Province, and belongs to the hinterland of Sanjiang Plain and the south bank of the lower Songhua River. This area has a temperate, semi-humid continental monsoon climate and a cold, long winter, with mean annual temperature of 2.5°C, mean annual precipitation of 476 mm, and a 133-day frost-free period. In both counties, the rice is transplanted in mid-May and harvested in late September. The rice field is ploughed deeply at the end of October, after which the rice root residues are left in the soil. The field lies fallow in the other months. The field is waterlogged during the entire growth season.

Soil samples were taken from five paddy fields in each county. The basic chemical-physical properties of soil samples were shown in Table 1. The criteria for selecting the fields were as follows: similar rice cropping systems (successive cropping, one crop per annual, growth period extends from the middle of May to late September), cultivation history (over 50 years), fertilization management (chemical and not organic fertilizer was used; the annual application rate of N, P, and K fertilizer was similar in the same paddy field; the fertilizer application rate was: N 90 to 120 kg hm^-2^, P_2_O_5_ 45 to 60 kg hm^-2^, K_2_O 45 to 75 kg hm^-2^), terrain (the slope steepness of sample location is less than 1°), and different soil fertility (based primarily on soil fertility, but also taking rice yield of past decades into account). Soil investigation and sample collection was conducted in October 2011 before ploughing. The soil sampling depth was 0–20 cm. To obtain the soil samples, three typical 500 m × 500 m sampling areas in each paddy field were delineated, and 5 sub-samples were randomly obtained from each sampling area. The samples were taken in between two rice plants to avoid sampling the roots, and the 5 sub-samples were combined to form one soil sample. Three replicated samples were collected in each paddy field, and the average of the determination values of the three replicates represented the result for each paddy field.

**Table 1:** Chemical-physical properties^†^ of experimental soils. Soil No. 1–1 to 1–5 were from Qingan County; soil No. 2–1 to 2–5 were from Huachuan County.

Soil No.	Location	pH	OC	TN	Fe	Clay	Silt	Sand	CEC	Ca	Initial N			
											NO_3_^−^-N	NH_4_^+^-N	SON	TSN
			g kg^-1^						cmol kg^-1^		mg kg^-1^			
1–1	46°58'N, 127°55'E	6.52	38.52	2.68	11.42	367.7	239.7	392.7	36.99	18.77	4.67	4.34	4.65	13.66
1–2	46°58'N, 127°48'E	6.30	33.42	2.47	12.03	366.3	252.0	381.7	32.45	18.52	4.42	4.91	3.91	13.24
1–3	46°53'N, 127°28'E	6.12	26.01	1.93	7.00	393.2	147.5	459.4	29.06	14.70	1.13	4.93	3.60	9.66
1–4	46°57'N, 127°25'E	5.96	20.76	1.61	8.17	371.7	196.1	432.2	25.67	14.46	5.65	6.50	5.17	17.32
1–5	47°01'N, 127°27'E	5.66	16.13	1.39	8.04	320.8	166.9	512.3	20.99	7.62	3.34	4.94	2.42	10.70
2–1	46°54'N, 130°36'E	5.65	22.61	1.76	4.29	273.2	177.2	549.6	22.03	9.39	4.85	4.29	5.09	14.23
2–2	46°53'N, 46°53'E	5.73	19.62	1.42	4.31	227.6	147.6	624.8	18.29	7.60	4.80	4.12	4.40	13.32
2–3	46°51'N, 130°31'E	5.98	17.90	1.14	6.15	313.9	241.6	444.6	31.28	9.30	4.64	3.96	3.21	11.81
2–4	46°52'N, 130°31'E	5.96	15.10	1.33	5.18	308.3	235.1	456.6	35.36	8.28	4.01	3.92	5.03	12.96
2–5	46°51'N, 130°32'E	5.94	12.04	0.90	4.45	298.5	85.4	616.1	18.98	8.62	2.06	4.29	2.96	9.31

OC = organic carbon; TN = total nitrogen; Fe = ammonium oxalate extractable iron; CEC = cation exchange capacity; Ca = exchangeable calcium; SON = soluble organic nitrogen; TSN = total soluble nitrogen.

### Methods

The organic N mineralization incubation experiment was carried out under waterlogged incubation [37, 38]. Fifteen grams of air-dried soil and 15 g of quartz sand were both sieved through a 2-mm screen into a 100-ml polyvinyl chloride incubation tube with three replicates. Leaching before waterlogged incubation and leaching during waterlogged incubation were then performed as described below. (1) Initial leaching before waterlogged incubation: Thirty millilitres of distilled water was added to the tube, the tube was sealed with a cap, and the soils were leached once. Thereafter, 30 ml of 1 M KCl solution was added and the soils were leached twice. Finally, 30 ml of distilled water was added and the samples were leached twice. During each leaching, the contents of the tubes were stirred after the liquid was added and the tubes were centrifuged for 10 minutes at 2800 × *g* (TDL-5M, XiangYi Instrument Inc.; China). Supernatants were collected in a 200-ml volumetric flask and distilled water was added to achieve a volume of 200 ml. The mineral N (NH_4_^+^-N and NO_3_^−^-N) and TSN was determined in the initial leachates. (2) Leaching during waterlogged incubation: After initial leaching, 30 ml of distilled water was added and the tube was sealed with a cap, and then the soil was under waterlogged incubation at 30°C in a controlled temperature cabinet for 161 days (a 2: 1 water: soil ratio was maintained during incubation). Then the soils were leached following the method described above on days 4, 7, 14, 21, 35, 49, 70, 91, 126, and 161 during the incubation. After each leaching, the soil was incubated under the same conditions, and the NH_4_^+^-N and TSN contents in the leachates were determined.

The NH_4_^+^-N and NO_3_^−^-N content in the initial leachates and NH_4_^+^-N during incubation was measured with Auto Analyzer 3 (Bran+Luebbe; Germany). The TSN in the initial leachates and during incubation was first oxidized with alkaline potassium persulphate solution (0.15 M NaOH and 3% K_2_S_2_O_8_) at approximately 123 ± 12°C for 30 minutes, and then measured with Auto Analyzer 3 [29]. The initial SON content was calculated as the difference between initial TSN and initial inorganic N (NH_4_^+^-N and NO_3_^−^-N) (Table 1). The SON content during incubation was calculated as the difference between TSN and NH_4_^+^-N.

Cumulative mineralised N (NH_4_^+^-N and TSN) was simulated by three models. The three models used in the model fitting are described below.

The single first-order kinetics model:

$$N_{t}=N_{0}\left(1-e^{\left(-k_{1}t\right)}\right)$$ (1)

where *N*_*t*_ is the cumulative amount of mineralised N over time *t*, *N*_0_ is defined as the potentially mineralised N, *k*_1_ is the first-order rate constant of mineralization of *N*_0_, and *t* is the incubation time [32].

The double first-order kinetics model:

$$N_{t}=N_{D}\left(1-e^{\left(-k_{D}t\right)}\right)+N_{R}\left(1-e^{\left(-k_{R}t\right)}\right)$$ (2)

where *N*_*D*_ and *k*_*D*_ represent the potentially mineralisable N and the first-order rate constant of mineralization of the readily available fraction, respectively; and *N*_*R*_ and *k*_*R*_ are the potentially mineralisable N and the first-order rate constant of mineralization of the resistant matter fraction, respectively, and *t* is the incubation time [33].

The mixed first-order and zero-order kinetics model:

$$N_{t}=N_{F}\left(1-e^{\left(-k_{F}t\right)}\right)+k_{0}t$$ (3)

where *N*_*F*_ and *k*_*F*_ are the potentially mineralizable N and the first-order rate constant of mineralization of the N pool made available after a drying and rewetting event, respectively, *k*_0_ is the zero-order rate constant of mineralization of the resistant N pool, and *t* is the incubation time [36].

Organic C and total N was measured with the C/H/O/N/S Elemental Analyzer Vario EL III (Elementar Company; Germany) (Table 1). Soil pH was measured using a pH510 glass electrode using a 2.5: 1 water: soil ratio (Eutech Instruments; Singapore). The CEC, exchangeable calcium (Ca), and ammonium oxalate extractable iron (Fe) were measured using the method described by Lu, Li [29].

Results in tables and figures are the average of three replicates expressed on an oven-dry weight basis. Mineralization parameters were estimated from the cumulative amounts of NH_4_^+^-N in leachates and TSN over waterlogged incubation time via non-linear regression, calculated separately for all three models. All data were subjected to ANOVA and then tested using F statistic values. A probability level of *P* < 0.05 was considered significant for *F* statistic values and LSD comparisons. Correlations between cumulative mineralised N (NH_4_^+^-N and TSN) and OC, TN, pH, and exchangeable Ca were determined using Pearson product-moment correlations. The significant correlations (*P* < 0.05) (positive or negative) were determined to be relevant for further consideration using linear regression analyses. The regression analysis was conducted using the stepwise multiple regression procedure, and a probability level of *P* < 0.05 was considered a significant regression. All statistical analyses were conducted with SPSS Windows version 19.0 (SPSS Inc., Chicago, USA). All the data shown in all figures, and supporting all main results, is publicly available.

## Results

### Cumulative NH_4_^+^-N, TSN, and SON during incubation

Waterlogged incubation is widely used to analyse soil N mineralization and the N supply index. The inhibition of bacterial nitrification activities under waterlogged incubation prevents the NH_3_ volatilization loss and NO_3_^−^-N denitrification loss, and creates an appropriate environment for soil organic N mineralization, of which the end product is NH_4_^+^-N [38]. Li, Han [3] found that the mineralised N was mainly NH_4_^+^-N, although some NO_3_^−^-N was found on days 0 and 1 under the incubation conditions. In the study, NO_3_-N content of initial leaching was 1.13–5.65 mg kg^-1^ and 2.06–2.855 mg kg^-1^ in Qingan and Huachuan country, respectively (Table 1). After 4 days under the incubation conditions, NO_3_-N content reduced, which was 0.10–1.25 mg kg^-1^ and 0.22–1.18 mg kg^-1^, respectively. After 7 days, NO_3_-N content couldn’t be test in both site. Therefore, the amount of N mineralization was only represented by NH_4_^+^-N content.

**Fig 1:**
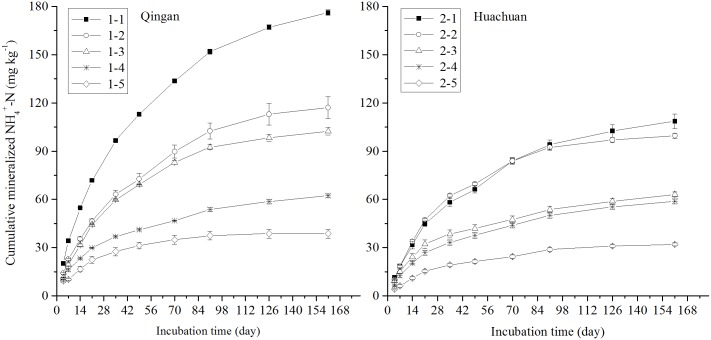
Cumulative mineralised NH4+-N during 161 days under waterlogged incubation for five soil samples from both Qingan County and Huachuan County in Mollisols area of Northeast China. Soil No. 1–1 to 1–5 were from Qingan County; soil No. 2–1 to 2–5 were from Huachuan County.

The cumulative N mineralization curves of soil samples from Qingan and Huachuan counties were similar (Fig 1). Cumulative NH_4_^+^-N increased quickly during the first 35 days, and then remained steady during days 35–91, and slowed down after 91 days. After 161 days of incubation, the cumulative NH_4_^+^-N content was 99.3 mg kg^-1^ (38.7–176.1 mg kg^-1^) and 72.4 mg kg^-1^ (32.0–108.6 mg kg^-1^), and the ratio of NH_4_^+^-N to TN was 4.7% (2.8%–6.6%) and 5.3% (3.6%–7.0%) in the soil samples from Qingan County and Huachuan County, respectively. The average cumulative NH_4_^+^-N content was 85.9 mg kg^-1^, and the ratio to TN was 5.0% in the ten soil samples.

**Fig 2:**
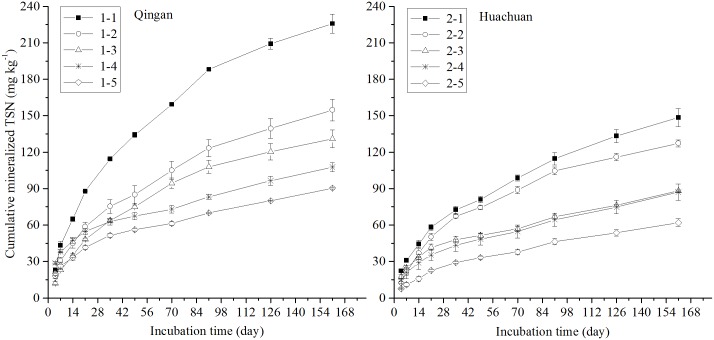
Cumulative mineralised total soluble N (TSN) during 161 days under waterlogged incubation for five soil samples from both Qingan County and Huachuan County in Mollisols area of Northeast China. Soil No. 1–1 to 1–5 were from Qingan County; soil No. 2–1 to 2–5 were from Huachuan County.

The N mineralization capacity significantly increased when SON was included in the soil samples from the two counties (Fig 2). When SON was considered, the cumulative TSN was 141.8 mg kg^-1^ (90.4–225.8 mg kg^-1^), representing 6.9% (6.2%–8.4%) of TN in Qingan County, and 102.6 mg kg^-1^ (62.0–148.5 mg kg^-1^), representing 7.7% (6.5%–8.9%) of TN in Huachuan County. The cumulative TSN was 122.2 mg kg^-1^, and the ratio to TN was 7.3% in the ten soil samples.

The SON was calculated as the difference between TSN and NH_4_^+^-N. The average cumulative SON was 42.5 mg kg^-1^ (28.6–51.6 mg kg^-1^) and 30.2 mg kg^-1^ (25.2–39.9 mg kg^-1^), the ratio to cumulative TSN was 33.4% (21.9%–51.7%) and 31.6% (21.7%–48.4%), and the ratio to cumulative NH_4_^+^-N was 58.5% (27.3%–133.4%) and 49.2% (27.8%–93.7%) for Qingan County and Huachuan County, respectively. The average cumulative SON was 36.3 mg kg^-1^, and the ratio to cumulative TSN or NH_4_^+^-N was 32.5% and 54% in the ten soil samples, respectively. Thus, after an incubation of 161 days, the amount of soil N mineralization was lower and the ratio of mineralization N to TN was slightly higher in Huachuan County as compared to Qingan County. The cumulative TSN (with SON) was 1.5 times the cumulative NH_4_^+^-N (without SON) in both counties. This is because the amount of total N in Qingan is higher than that in Huachuan.

**Fig 3:**
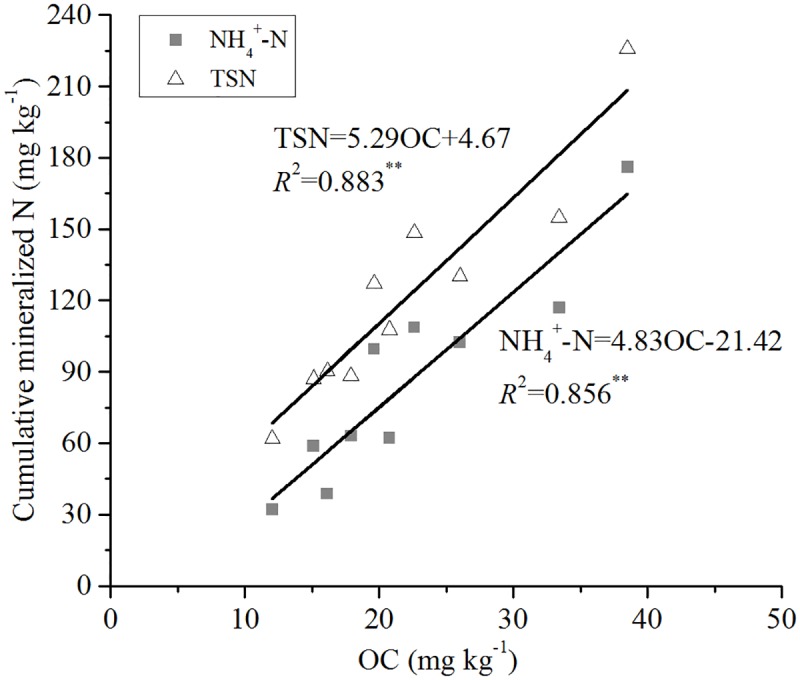
Relationship between cumulative mineralised N (NH4+-N and TSN) during 161 days and soil OC.

**Fig 4:**
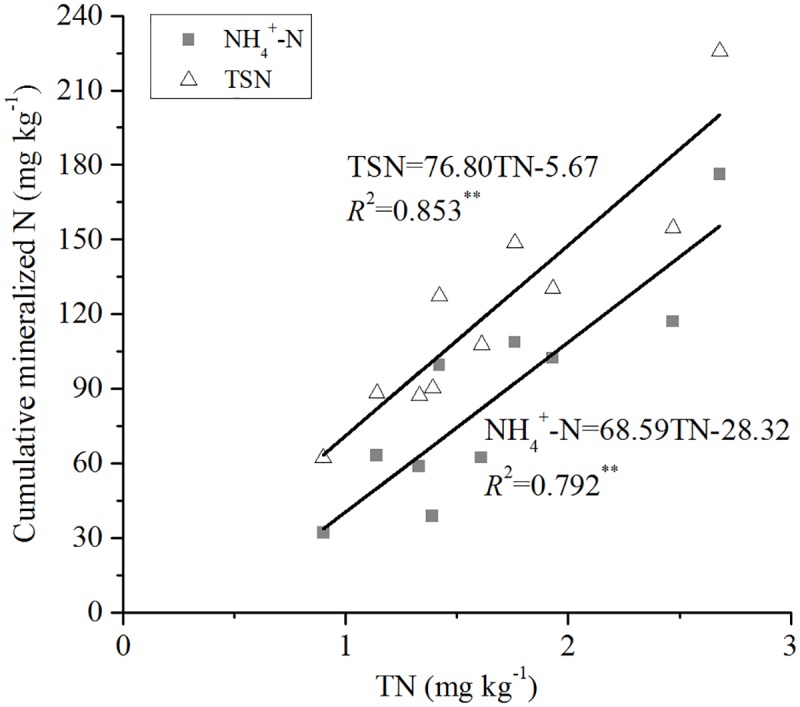
Relationship between cumulative mineralised N (NH4+-N and TSN) during 161 days and soil TN.

**Fig 5:**
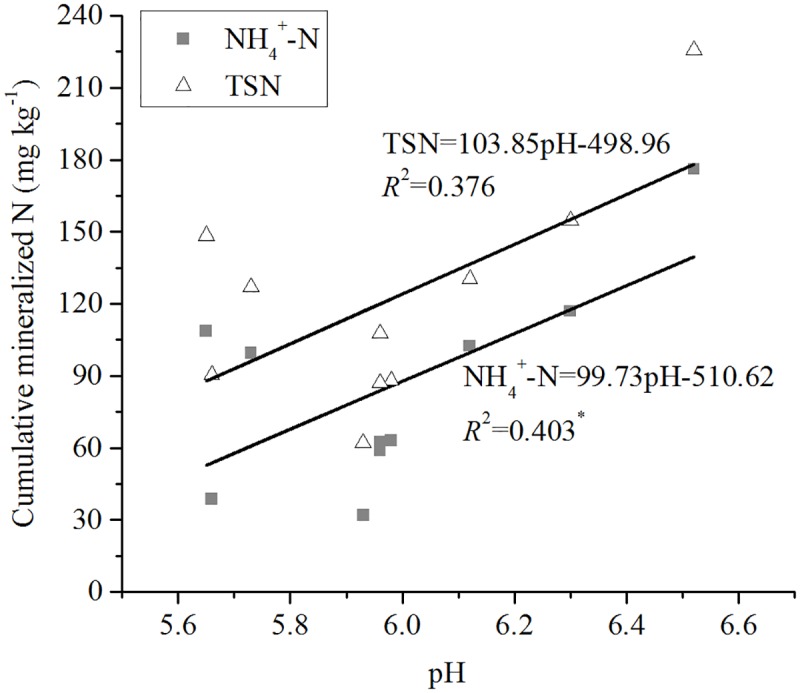
Relationship between cumulative mineralised N (NH4+-N and TSN) during 161 days and soil pH.

**Fig 6:**
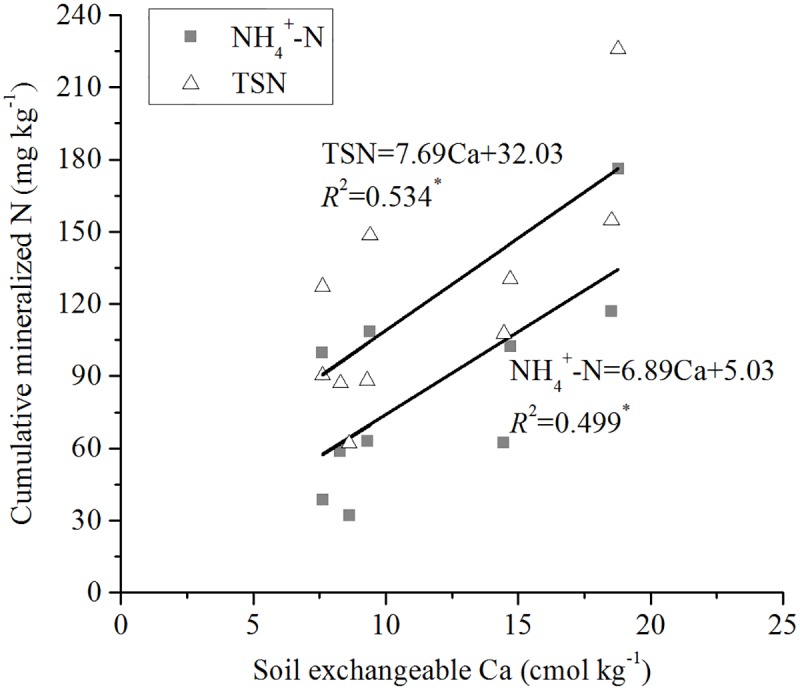
Relationship between cumulative mineralised N (NH4+-N and TSN) during 161 days and soil exchangeable Ca.

Cumulative NH_4_^+^-N leached in the soils was significantly (*P* < 0.05) positively correlated with OC (*R*^2^ = 0.856) (Fig 3), TN (*R*^2^ = 0.792) (Fig 4), pH (*R*^2^ = 0.403) (Fig 5), and exchangeable Ca (*R*^2^ = 0.499) (Fig 6). Mineral TSN leachate in the soils was significantly (*P* < 0.05) positively correlated with OC (*R*^2^ = 0.883) (Fig 3), TN (*R*^2^ = 0.853) (Fig 4), and exchangeable Ca (*R*^2^ = 0.534) (Fig 6). However, it was not significantly (*P* > 0.05) correlated with C/N ratio, CEC, extractable Fe, clay, and sand.

Stepwise multiple regression analyses of the mineralised NH_4_^+^-N with various combinations of soil characteristics showed that 98.3% (*R*^2^ = 0.983) of the variability in mineralised NH_4_^+^-N was accounted for by the following eq (4), and the most variability (*R*^2^ = 0.998) in the mineralised NH_4_^+^-N was accounted for by the subsequent regression eq (5).

$${\text{Mineralised NH}}_{4}{}^{+}\text{-N}=-10.95+8.02\text{OC}-6.73\text{Fe}-2.87\text{Ca}\left(R^{2}=0.983\right)$$ (4)

$$\begin{array}{ll} {\text{Mineralised NH}}_{4}{}^{+}-\text{N}= & 529.96+26.85\text{OC}+0.93\text{CEC }-274.44\text{TN }-25.44\text{C}/\text{N}\\ & -\;32.67\text{pH }-0.88\text{clay}-6.61\text{Fe }\left(R^{2}=0.998\right) \end{array}$$ (5)

Stepwise multiple regression analyses of the mineralised TSN with various combinations of soil characteristics showed that 94.8% (*R*^2^ = 0.948) of the variability in mineralised TSN was accounted for by the following eq (6), and the most variability (*R*^2^ = 0.999) in the mineralised TSN was accounted for by the subsequent regression eq (7).

$$\text{Mineralised TSN}=11.11+8.18\text{OC}-6.03\text{Ca}\left(R^{\text{2}}=0.948\right)$$ (6)

$$\begin{array}{ll} \text{Mineralised TSN}= & 1755.59+59.34\text{OC}+1.95\text{CEC}+4.69\text{Ca}-733.71\text{TN}\\ & -67.83\text{C/N}-148.41\text{pH}-0.80\text{clay}-3.35\text{Fe }\left(R^{2}=0.999\right) \end{array}$$ (7)

### Comparison of organic N mineralization process model simulations

**Fig 7:**
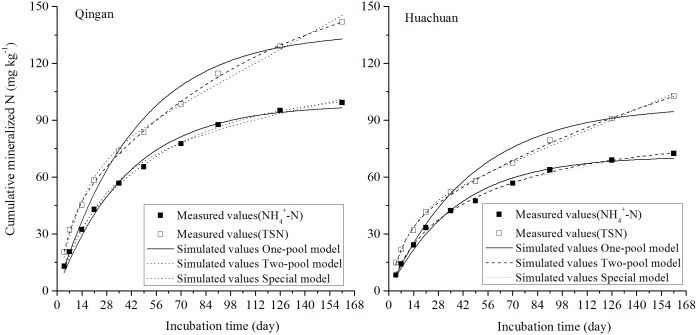
Average measured and simulated N mineralization curves of ten soil samples.

The simulation of the curves representing changes in average cumulative NH_4_^+^-N and TSN in the three models during the incubation of soil samples from the two counties is shown in Fig 7. The simulation values of the double first-order kinetics model and the mixed first-order and zero-order kinetics model simulated the measured values of N mineralization, whereas the simulation values of the single first-order kinetics model were lower than the measured values in the early and later periods of incubation and were higher than the values measured in the middle period. The simulation results of the models for cumulative NH_4_^+^-N and TSN are shown in Tables 2 and 3.

**Table 2:** Model parameters and root mean square errors (RMSE) of single first-order kinetics, double first-order kinetics, and mixed first-order and zero-order kinetics models simulated to cumulative NH4+-N data from samples. Soil No. 1–1 to 1–5 were from Qingan County; soil No. 2–1 to 2–5 were from Huachuan County.

Soil No.	Single first-order kinetics				Double first-order kinetics^†^						Mixed first-order and zero-order kinetics				
	*N*_0_	*k*_1_	*R*MSE	*F* value	*N*_*D*_	*k*_*D*_	*N*_*R*_	*k*_*R*_	*R*MSE	*F* value	*N*_*F*_	*k*_*F*_	*k*_0_	*R*MSE	*F* value
	mg kg^-1^	day^-1^	mg kg^-1^		mg kg^-1^	day^-1^	mg kg^-1^	day^-1^	mg kg^-1^		mg kg^-1^	day^-1^	mg kg^-1^ day^-1^	mg kg^-1^	
1–1	175.3	0.023	11.4	855.0***	30.4	0.120	160.5	0.015	0.6	6017.7***	114.2	0.039	0.404	4.4	1193.5***
1–2	118.9	0.022	5.4	814.5***	13.6	0.200	113.3	0.016	1.0	1650.3***	80.4	0.034	0.245	4.1	578.4***
1–3	102.5	0.025	1.6	2173.2***	11.2	0.125	95.0	0.020	0.5	2543.3***	83.4	0.032	0.126	1.1	1684.4***
1–4	58.6	0.030	5.0	198.0***	20.2	0.125	50.7	0.011	0.3	1448.6***	34.4	0.069	0.184	0.9	592.0***
1–5	38.0	0.041	0.9	446.8***	4.5	4.549E	34.6	0.032	0.3	597.8***	32.6	0.051	0.044	0.8	272.8***
2–1	109.5	0.022	3.7	1042.7***	16.2	0.109	101.5	0.015	1.5	989.1***	75.2	0.033	0.218	2.5	843.3***
2–2	99.5	0.028	1.6	2095.5***	20.7	0.068	82.2	0.021	1.5	901.0***	86.7	0.033	0.088	1.5	1237.0***
2–3	58.5	0.032	4.8	210.2***	28.3	0.087	51.8E	0.007E	0.4	1067.2***	36.1	0.068	0.174	0.5	1124.3***
2–4	56.7	0.026	2.8	358.3***	18.9	0.091	49.5	0.010	0.3	1339.2***	33.5	0.054	0.165	0.6	904.7***
2–5	31.3	0.027	0.7	460.4***	10.1	0.085	25.3	0.013	0.2	497.3***	21.0	0.047	0.074	0.3	528.2***

^†^ Simulating values with “E” means that the simulated values did not agree with real values.

*** Significant at the 0.001 probability levels.

**Table 3:** Model parameters and root mean square errors (RMSE) of single first-order kinetics, double first-order kinetics, and mixed first-order and zero-order kinetics models simulated to cumulative TSN data from samples. Soil No. 1–1 to 1–5 were from Qingan County; soil No. 2–1 to 2–5 were from Huachuan County.

Soil No.	Single first-order kinetics				Double first-order kinetics^†^						Mixed first-order and zero-order kinetics				
	*N*_0_	*k*_1_	*R*MSE	*F* value	*N*_*D*_	*k*_*D*_	*N*_*R*_	*k*_*R*_	*R*MSE	*F* value	*N*_*F*_	*k*_*F*_	*k*_0_	*R*MSE	*F* value
	mg kg^-1^	day^-1^	mg kg^-1^		mg kg^-1^	day^-1^	mg kg^-1^	day^-1^	mg kg^-1^		mg kg^-1^	day^-1^	mg kg^-1^ day^-1^	mg kg^-1^	
1–1	226.5	0.020	29.3	531.3***	43.2	0.116	219.2	0.011	4.0	1510.0***	119.6	0.044	0.687	10.6	5796.7***
1–2	154.2	0.019	23.4	286.6***	32.6	0.136	165.8E	0.008E	1.7	1574.1***	64.3	0.062	0.583	5.3	693.0***
1–3	133.8	0.019	6.4	848.2***	18.0	0.131	131.0	0.012	1.2	1789.4***	74.6	0.036	0.361	3.1	954.5***
1–4	89.9	0.044	52.8	30.0**	45.7	0.208	232.5E	0.002E	1.0	785.3***	48.0	0.191	0.377	0.9	1136.0***
1–5	79.6	0.032	24.5	61.3***	31.8	0.180	102.1E	0.005E	1.8	364.8***	39.0	0.129	0.325	2.0	461.4***
2–1	145.3	0.020	32.9	170.7***	35.3	0.156	173.9E	0.007E	1.3	1719.4***	55.4	0.085	0.600	4.1	760.1***
2–2	125.9	0.021	9.6	511.8***	38.4	0.072	123.5E	0.008E	1.8	1092.4***	66.1	0.046	0.389	2.1	1247.9***
2–3	76.4	0.031	27.0	51.0***	35.7	0.141	3924.9E	0.000E	0.8	810.6***	35.8	0.140	0.325	0.6	1419.1***
2–4	79.9	0.022	20.0	78.8***	28.0	0.147	214.4E	0.002E	0.5	1353.6***	30.7	0.128	0.352	0.5	1870.4***
2–5	61.9	0.017	4.7	220.2***	18.3	0.086	133.7E	0.002E	0.6	718.6***	21.7	0.072	0.253	0.5	1145.9***

^†^ Simulating values with “E” means that the simulated values did not agree with real values.

**, *** Significant at the 0.01 and 0.001 probability levels, respectively.

The single first-order kinetics model. When simulations with the single first-order kinetics model were performed, the *N*_0_ increased significantly and *k*_1_ decreased slightly in the cumulative TSN with SON simulation, compared to the cumulative NH_4_^+^-N without SON simulation (Tables 2 and 3). Regardless of whether or not SON was included, there was a significantly (*P* < 0.01) positive relationship between *N*_0_ and the cumulative mineralised N during 161 days or TN. In the cumulative NH_4_^+^-N without SON simulation, the correlation coefficients (*P* < 0.01) between *N*_0_ or the cumulative NH_4_^+^-N during 161 days and TN were 0.925 and 0.890, respectively, in ten soil samples from both counties. In the cumulative TSN with SON simulation, the correlation coefficients (*P* < 0.01) between *N*_0_ or the cumulative TSN during 161 days and TN were 0.940 and 0.924, respectively, in ten soil samples from both counties. However, for some soils, the value of *N*_0_ was lower than the actual value of the cumulative mineralised N during 161 days, indicating a shortcoming of the single first-order kinetics model when simulating the N mineralization process of paddy soil under waterlogged incubation.

The double first-order kinetics model. When simulating with the double first-order kinetics model, the mineralization rate of readily available N fraction (*k*_*D*_) was significantly higher than that of the resistant matter fraction (*k*_*R*_) (Tables 2 and 3). Compared to the cumulative NH_4_^+^-N simulation, in the cumulative TSN simulation, *N*_*D*_ clearly increased with an average of 18.3 mg kg^-1^ and 12.3 mg kg^-1^, and the ratio to cumulative SON had mean values of 54.6% and 37.8% in the soils from Qingan County and Huachuan County, respectively. The increasing amount of *N*_*D*_ was significantly and positively correlated with cumulative SON in the ten soil samples (*r* = 0.732, *P* < 0.05), which indicates that SON is an important contributor to the decomposable N pool. When SON was considered, the mineralization rate of the readily available N fraction decreased slightly when soil TN content was over 1.93 g kg^-1^, whereas the rate increased slightly when soil TN content was less than 1.76 g kg^-1^. These findings may be related to the ratio of SON to TN produced during the incubation, with the average SON derived from mineralization being 16.2 mg g^-1^ when soil TN content was over 1.93 g kg^-1^, and 27.9 mg g^-1^ when soil TN content was less than 1.76 g kg^-1^. Regardless of whether or not SON was included, *N*_*D*_ and *N*_*R*_ were not closely correlated with the cumulative mineralised N during 161 days and TN. The ratio of (*N*_*D*_ + *N*_*R*_) to the cumulative mineralised N during 161 days of soil sample 2–3 from Huachuan County was 1.4 when SON was considered, whereas the ratios of soil samples 1–4 and 1–5 from Qingan County were 3.0 and 1.7 without SON, respectively. The ratios of (*N*_*D*_ + *N*_*R*_) to the cumulative mineralised N during 161 days of soil samples from Huachuan County increased (up to 51.8), which did not simulate the actual mineralization process. These results suggest that the simulation effect of a double first-order kinetics model for testing the soil N mineralization process is relatively poor, which was particularly apparent when SON was included. The *N*_*D*_, *N*_*R*_ and *k*_*D*_, *k*_*R*_ did not reflect the mathematical simulation results for abnormal values, and could not accurately represent the soil N mineralization potential and rate.

The mixed first-order and zero-order kinetics model. Compared to the cumulative NH_4_^+^-N simulation, in the cumulative TSN simulation, the average value of *N*_*F*_ of soil samples from Qingan County increased slightly, whereas the average *N*_*F*_ decreased by approximately 8.56 mg kg^-1^ in the soil samples from Huachuan County. The *k*_*F*_ of the soil samples from both areas increased by nearly two times, whereas *k*_0_ increased by 2.3 and 2.7 times in the soil samples from Qingan County and Huachuan County, respectively, suggesting that SON does not contribute to the mineralization N pool made available after a drying and rewetting event. In the cumulative NH_4_^+^-N without SON simulation, the correlation coefficients (*P* < 0.01) between *N*_*F*_ and the cumulative NH_4_^+^-N during 161 days or TN were 0.958 and 0.825, respectively, and the correlation coefficients (*P* < 0.01) between *k*_0_ and the cumulative NH_4_^+^-N during 161 days or TN were 0.827 and 0.795, respectively, in the ten soil samples from both counties. In the cumulative TSN with SON simulation, the correlation coefficients (*P* < 0.01) between *N*_*F*_ and the cumulative TSN during 161 days or TN were 0.954 and 0.868, respectively, and the correlation coefficients (*P* < 0.01) between *k*_0_ and the cumulative TSN during 161 days or TN were 0.927 and 0.876, respectively, in the ten samples examined, indicating that *N*_*F*_ and *k*_0_ showed a more significant correlation with TN.

Based on the results from the mixed first-order and zero-order kinetics model simulation, the simulation curve presented as a first-order growth model in the first 35 days. During these initial 35 days, the average mineralised N obtained from the first-order growth model was 87.9% and 75.7% of the average cumulative mineralised N amount in the cumulative NH_4_^+^-N and TSN simulation, respectively, of the ten soil samples. After 35 days, the mineralization N amount from the mineralization N pool made available after a drying and rewetting event decreased over incubation time, whereas the mineralization N amount of the resistant N pool increased. In addition, the curve showed a linear growth trend, which was particularly obvious in the cumulative TSN (with SON) simulation. The mixed first-order and zero-order kinetics model showed a precise simulation in the first 91 days of incubation, and the simulated value gradually increased after 91 days because of the decrease in the mineralization rate. At the end of the incubation, the simulated value was slightly higher than the measured value, indicating that the mixed first-order and zero-order kinetics model could accurately simulate the N mineralization of paddy soil in Mollisols area of Northeast China under 161 days of waterlogged incubation.

The estimated standard errors and *F* statistic values of the model simulations are shown in Tables 2 and 3. Compared to the cumulative NH_4_^+^-N simulation, in the cumulative TSN simulation, the estimated standard error increased of the three models, the *F* statistic values decreased slightly in the single first-order kinetics and double first-order kinetics models, and increased slightly in the mixed first-order and zero-order kinetics model. These findings suggested that SON had a definite effect on model simulation. Regardless of whether or not SON was included, this comprehensive analysis of the simulative consequence and the actual N mineralization process suggested that the mixed first-order and zero-order kinetics model was superior to the single first-order kinetics and double first-order kinetics models. Therefore, the mixed first-order and zero-order kinetics model is more appropriate to describe the N mineralization process of paddy soil in Mollisols area of Northeast China.

## Discussion

### The effect of soil characteristics on N mineralization

In a study of 39 diverse Philippine wetland rice soils, the mineralization of soil organic N under waterlogged incubation was highly positively correlated with OC and TN, and was negatively correlated with the C/N ratio of the soils [39]. Mineral N released in the soils was significantly (*P* < 0.01) positively correlated with OC and TN, but was not significantly (*P* > 0.05) correlated with the extractable Fe and C/N ratios [40]. Our results support the findings of Narteh and Sahrawat [40]. Ammonium N released in soil samples was poorly correlated with soil OC or TN. The differences in soil organic matter quality related to intensive cropping in submerged soil influences the relationships between mineralised N and soil organic matter [41]. Recent studies have shown that the stabilization of applied organic matter and fertilizer N in the humic fractions of soil organic matter under waterlogged conditions of lowland rice soils influences the mineralization of organic matter and release of mineral N [42, 43]. Such effects may be attributed to the accumulation of lignin, and the phenolic subunits of lignin that are resistant to degradation in the absence of oxygen contribute to the changes in the quality of organic matter in submerged soil [44, 45]. In addition, the formation of stable complexes of humic substances with cations such as ferrous Fe protects organic matter from degradation [46, 47].

It was found that other soil characteristics, such as pH, clay, and CEC were not significantly (*P* > 0.05) correlated with ammonium production; however, multiple regression analyses showed that the inclusion of these characteristics had significant effects on the release of mineral N in the soils [39]. Mineral N released in the soils was significantly (*P* < 0.01) positively correlated with clay, CEC, and pH [40]. The chief factors responsible for poor N mineralization were exchangeable H and the abundance of Al and Fe gels [48]. Our results also showed that the mineralised N was correlated with pH and exchangeable Ca (Figs 5 and 6), but not correlated with CEC and Fe. Fe was correlated with OC, TN, pH, and sand (Table 4). Although simple correlation analyses showed that Fe was not correlated with mineralised N, stepwise regression showed that it had an effect on N mineralization. This finding may be explained by the availability of electron acceptors that affect organic matter oxidation and ammonium production or N mineralization in submerged soils [49]. For example, easily reducible Fe or Fe that participates in the Fe redox reactions in soils plays a dominant role in the oxidation of organic matter and the production of NH_4_^+^-N in submerged soils and sediments [49–52].

**Table 4:** Matrix of correlation coefficients (*r*) between OC, TN, pH, sand, Fe, and Ca of paddy soils.

Soil property^†^	OC	TN	pH	Sand	Fe
OC	--				
TN	0.98 ***	--			
pH	0.77 **	0.72 **	--		
Sand	-0.64 *	-0.65 *	-0.73 *	--	
Fe	0.77 **	0.80 **	0.76 *	0.76 *	--
Ca	0.90 **	0.88 **	0.87 **	0.87 **	0.86 **

^†^ OC = organic carbon; TN = total nitrogen; Fe = ammonium oxalate extractable iron; Ca = exchangeable calcium

*, **, *** Significant at the 0.05, 0.01 and 0.001 probability levels, respectively.

Some results illustrate that prediction ability of Ca on N mineralization was affected by other soil physicochemical properties, and this mechanism need further study. Cui, Liu [53] found that extractable Ca^2+^ content of paddy soil was higher than upland soils in topsoil of reclamation of coastal wetlands of Yangtze Estuary. Xu, Wang [54] reported that the extent of pH changed after addition of crop residues, low soil pH greatly inhibited nitrification, and calcium derived from the crop residues might have a direct impact on pH value changing. Moreover, pH was the main factor that affect the community and activity of microorganism [13, 15, 55], the existing research indicated that Ca influence on soil N transformation and mineralization through affecting pH or interacting with phosphorus [56]. In this study Ca was close correlated with OC, TN and pH etc. (*p* < 0.01) (Table 4), moreover, multiple regression analyses showed that Ca and other factors(e.g. OC, TN, pH, Sand and Fe) were common affecting soil accumulation mineralised NH_4_^+^-N.

### SON content in the paddy soil N mineralization process

Studies have demonstrated that mineralization incubation leachates contain a certain amount of SON when soils from farmland and forest are incubated, but the content and percentage of SON varies depending on soil type, land use, and incubation conditions [25, 26, 29]. In a study of ten major types of farmland soils on the Loess Plateau in China under waterlogged incubation and aerobic conditions, the average initial content of SON was 23.9 mg kg^-1^, and the ratio to the initial TSN or TN was 28.8% and 2.4%, respectively [29]. We studied ten soil samples under waterlogged incubation and found that the average content of initial SON was 4.0 mg kg^-1^, and the ratio to initial soil TSN or TN was 32.0% and 0.2%, respectively. These results indicate that soil initial SON varies depending on soil type and incubation condition, and the content of initial SON and the ratio to TN under waterlogged incubation is low.

Measuring only the quantity of inorganic N mineralization may underestimate the mineralization potential of paddy soil in Mollisols area of Northeast China. Smith, Schnabel [25] found that during 11 weeks of aerobic incubation, the average content of SON was 45.3 mg kg^-1^, and the ratio to TSN or TN was 41.5% and 7.6%, respectively. In a long-term located experiment studying the use of different fertilizers in the alkaline soil of Northern China [26], the SON content in non-fertilized soil was 8.5 mg kg^-1^, and the ratio to TSN or TN was 13.4% and 0.4%, respectively, during 19 weeks of aerobic incubation. For ten major types of farmland soils on the Loess Plateau in China under waterlogged incubation and aerobic conditions, the average content of SON was 28.8 mg kg^-1^, and the ratio to TSN and TN was 19.8% and 2.7%, respectively, under aerobic incubation [29]. However, the SON pool was almost the same as the inorganic N pool in leachates, the average SON content was 118.1 mg kg^-1^, and the ratio to TSN and TN was 46.6% and 11.1%, respectively, under waterlogged incubation [30]. We studied ten soil samples during 23 weeks of waterlogged incubation, and found that the average content of SON was 36.3 mg kg^-1^, and the ratio to TSN and TN was 29.7% and 2.1%, respectively. For ten major types of farmland soils on the Loess Plateau in China, during 31 weeks of waterlogged incubation and aerobic incubation, the SON content and percentage of TSN significantly increased under waterlogged incubation compared to leaching under aerobic incubation [29, 30]. These findings demonstrate that the SON released during the soil organic N mineralization process varies depending on soil type and incubation condition. Our study demonstrated that the SON content and the ratio to TN was low, but the ratio to SON was high under waterlogged incubation.

Currently, there are contrasting opinions about the degradation characteristics of soil SON and its contribution to soil mineral N. Smith [57] suggested that the degradation rate of SON is low and its contribution to soil mineral N is limited under aerobic or waterlogged incubation conditions. In contrast, Cookson and Murphy [28] found that when soil soluble organic matter was leached off the soil before incubating, the amount of mineralisable N decreased by 12%–22% and the N mineralization rate decreased by 17%–25%, suggesting that the contribution of SON to soil mineral N must be considered. Zhao, Cai [31] found that the UV280 value (the absorption value of soluble organic matter at 280 nm) and the HIXem value (Humification index, the ratio of the area at 435–480 nm long-wave domain to the area at 300–345 nm short wave domain) of soluble organic matter in leachates during incubation increased slightly compared to the initial values. This finding was attributed to the increase in relatively complex aromatic compounds in soluble organic matter and the resistant fraction. Therefore, further study on the degradation characteristics of SON in leachates and its contribution to soil mineral N is merited.

### Determining a suitable model of the mineralization process in paddy soil

That SON could reflect the organic N mineralization process. The waterlogged incubation method, first proposed by Waring and Bremner (1964), has been applied widely to study the N mineralization process in paddy soil owing to its convenience (it has no requirements to aerate or add water during the incubation period), and how closely it mimics the actual N mineralization process [3, 4, 6]. However, previous studies on the N mineralization process in paddy soil have focussed mainly on the inorganic N content, without considering SON mineralization. In this experiment, the cumulative SON was nearly half the cumulative inorganic N, and the linear correlation between TSN and OC or TN was more significant when including SON during incubation for 23 weeks.

Previous studies on organic N mineralization in paddy soil have confirmed that compared to the effective cumulative temperature model under waterlogged incubation, single first-order kinetics, double first-order kinetics, and mixed first-order and zero-order kinetics models were superior, with the mixed first-order and zero-order kinetics model showing the best fit to actual data [3–5]. In this study, regardless of whether or not SON was considered, the estimated standard error and F statistic values of model simulation (Tables 2 and 3) showed that the double first-order kinetics and mixed first-order and zero-order kinetics models were better than the single first-order kinetics model. However, when SON was considered, the estimated standard error of the single first-order kinetics model increased relatively more than that of the other two models, and *F* statistic values of the single first-order kinetics and double first-order kinetics models decreased slightly, whereas that of the mixed first-order and zero-order kinetics model increased slightly. Lu, Li [4] found that the mixed first-order and zero-order kinetics model could accurately simulate the N mineralization process of calcareous soil under waterlogged conditions, with and without SON, in ten types of major calcaric agricultural soils collected from the Loess Plateau. The parameter *N*_*F*_ of the mixed first-order and zero-order kinetics model was clearly related to crop N uptake, indicating that it could be used as an evaluation index of soil N supply capacity. In particular, Lu, Li [29] suggest that since the mineralisable N pool of the air-dry process was taken into consideration using the mixed first-order and zero-order kinetics model, it is more appropriate for waterlogged incubation experiments of air-dried soil. Confirming this perspective, the results of our study suggest that regardless of whether or not SON is considered, the mixed first-order and zero-order kinetics model could effectively describe the N mineralization process in waterlogged incubation for paddy soil. However, further in-depth analysis of the relationship between the parameter *N*_*F*_ of the mixed first-order and zero-order kinetics model and crop N uptake is required.

## Conclusions

This study resulted in two major conclusions that will be valuable in developing effective N fertilization strategies to increase productivity of paddy soil. Firstly, during the period of waterlogged incubation of paddy soil in Mollisols area of Northeast China, mineralization NH_4_^+^-N was significantly (*P* < 0.05) positively correlated with OC, TN, pH, and exchangeable Ca, and mineralization TSN was significantly (*P* < 0.05) positively correlated with OC, TN, and exchangeable Ca, but was not significantly (*P* > 0.05) correlated with C/N ratio, CEC, extractable Fe, clay, and sand. The ratio of cumulative SON to NH_4_^+^-N or TSN was 54.0% and 32.5%, respectively, and TSN showed a stronger linear correlation with OC or TN when considering SON. Therefore, SON should be considered during evaluations of N mineralization.

Secondly, the mixed first-order and zero-order kinetics model accurately simulated the N mineralization process of paddy soil in Mollisols area of Northeast China under waterlogged incubation, both with and without SON, based on the standard error estimates, *F* statistic values, actual significance of the simulative parameter, and simulation effects of estimated values and measured values of the three models.

In the further, study on the degradation characteristics of SON in leachates and its contribution to soil mineral N should be considered. In addition, further research should consider the relationship between the parameter *N*_*F*_ of the mixed first-order and zero-order kinetics model and crop N uptake.

## Supporting information

S1 Dataset Data for the Figs 1–7. Data of Figs 1–7 showed in the corresponding worksheet of Figs 1–7 supporting information.
(XLSX)
